# Evaluation of Gestational Diabetes Mellitus Risk in South Indian Women Based on *MTHFR* (C677T) and *FVL* (G1691A) Mutations

**DOI:** 10.3389/fped.2015.00034

**Published:** 2015-05-05

**Authors:** Imran Ali Khan, Noor Ahmad Shaik, Vasundhara Kamineni, Parveen Jahan, Qurratulain Hasan, Pragna Rao

**Affiliations:** ^1^Department of Genetics and Molecular Medicine, Kamineni Hospitals, Hyderabad, India; ^2^Department of Genetics, Vasavi Medical and Research Center, Hyderabad, India; ^3^Department of Genetics and Biotechnology, Osmania University, Hyderabad, India; ^4^Department of Genetic Medicine, Faculty of Medicine, King Abdulaziz University, Jeddah, Saudi Arabia; ^5^Department of Gynecology and Obstetrics, Kamineni Hospitals, Hyderabad, India; ^6^Department of Biochemistry, Kasturba Medical College, Manipal University, Manipal Karnataka, India

**Keywords:** gestational diabetes mellitus, *MTHFR*, *FVL*, C677T, G1691A

## Abstract

We aimed to scrutinize the extent to which single amino acid substitutions in the *MTHFR* and factor V Leiden (*FVL*) genes affect the risk of gestational diabetes mellitus (GDM) in pregnant women of South Indian descendant. This case–control study was implemented once the ethical approval has been obtained. Overall, 237 women were recruited in this study: 137 had been diagnosed with GDM and the remaining 100 women were used as normal controls or non-GDM. The diagnosis of GDM was confirmed with biochemical analysis, i.e., GCT and oral glucose tolerance tests. Five milliliters of peripheral blood was collected and used for biochemical and molecular analyses. DNA was isolated, and genotyping for *MTHFR* (C677T) and *FVL* (G1691A) mutations was performed using PCR–RFLP. FVL (G1691A) locus was not polymorphic in the investigated sample. There was no significant difference in the allele and genotype frequencies of C677T polymorphism between GDM and non-GDM women (*p* = 0.8892).

## Introduction

Gestational diabetes mellitus (GDM) is among the most common metabolic disorders affecting pregnant women characterized by glucose intolerance of variable severity, which is usually detected at the onset of or during pregnancy (1). GDM is treated by insulin or dietary modifications. It typically develops during the secondary trimester of pregnancy and resolves after the delivery (2). The risk of GDM women is intensified by advanced maternal age, ancestral disparities, obesity, and a family history of diabetes (3); however, the exact etiology is unknown due to limited knowledge of genetic factors (4). GDM patients are at increased risk of gestational hypertension, pre-eclampsia during pregnancy, and type 2 diabetes (T2DM), as well as metabolic syndromes in later life (5). Pregnant women with a history of GDM are at greater risk of developing T2DM, and a family history of diabetes predisposes pregnant women to GDM (6). Elevated fasting glucose and insulin levels are observed in women who do not have GDM but have a family history of the disease (7). Individuals of Asian descents have two- to sevenfold greater risk of developing GDM than their Caucasian counterparts in developed countries (8). Various epidemiological studies have reported an association between metabolic disorders and risk of developing T2DM or GDM, and numerous efforts have been made to identify pathogenic mutations in nuclear and mitochondrial genomes that are linked to both types of diabetes. Various susceptibility loci have been identified by linkage analyses and genome-wide association studies (1).

The methylenetetrahydrofolate reductase (*MTHFR*) gene has been mapped to chromosomal region 1p36.3 and comprises 11 exons encoding 5′, 10′-MTHFR (9), a key regulatory enzyme in folate metabolism that converts 5′,10′-MTHF to 5′-MTHF, the methyl donor for the remethylation of homocysteine to methionine (10). C677T is a common missense mutation in the gene that results in the substitution of alanine for valine at amino acid position 222. The A222V mutation is associated with a 50% reduction in MTHFR enzymatic activity, which increases plasma homocysteine and decreases plasma folic acid concentrations (11, 12). The *factor V Leiden (FVL)* gene is involved in the blood coagulation; G1691A mutation results in the substitution of guanine with arginine at the amino acid position 506, yielding a defective FVL protein that is unable to interact with the activated protein C, which was associated with increased coagulation activity and susceptibility to thromboembolism in previous studies. This mutation has also been linked to increased thrombosis risk and mortality in women following delivery/pregnancy (13, 14). Numerous studies have found an association between complications during pregnancy and inherited thrombophilias caused by mutations in *MTHFR* and *FVL* (15–17). However, there have been no studies to date addressing the potential role of *MTHFR* and *FVL* mutations in the development of GDM in the Indian population. The objective of this study was to investigate the extent to which single amino acid substitutions in the *MTHFR* and *FVL* genes affect the risk of GDM in pregnant women of South Indian descent.

## Materials and Methods

### Ethics statement

The study protocol was approved by the institutional ethics committee at Kamineni hospitals. Written, informed consent was obtained from all subjects, who were recruited by a clinical research midwife.

### Study subjects

This study was carried out in South Indian population from 2007 to 2011. Study subjects were recruited from Kamineni and Muslim maternity hospitals, Hyderabad. A total of 237 pregnant women participated in the study; of these, 137 had GDM, and 100 had normal glucose levels during pregnancy (i.e., the non-GDM group). A diagnosis of GDM was made by analyzing 3 mL of serum samples obtained from each subject; an additional 2 mL of blood was collected and stored in ethylenediamine tetraacetic acid (EDTA) in order to screen for *MTHFR* and *FVL* mutations. Among GDM patients, 58.4% had a family history of T2DM. Screening and management of diabetes during pregnancy were performed by qualified gynecologists according to guidelines set by the American Diabetes Association (18).

### Clinical and biochemical analyses

Gestational diabetes mellitus cases were identified using the glucose challenge test between weeks 24 and 28 of gestation; 50 g of glucose was administered to pregnant women with fasting plasma glucose levels exceeding 130 mg/dL. The oral glucose tolerance test (OGTT) was then performed using 100 g of glucose following an overnight fast and 3 days on an unrestricted diet. Fasting plasma samples were drawn 1–3 h after glucose administration. In this study, GDM cases were defined as those whose glucose levels met or exceeded threshold values described by Khan et al. (19). Women diagnosed with T1DM, T2DM, or any other form of diabetes before pregnancy, were excluded from the study. Body mass index (BMI) was calculated as weight/height^2^ (kg/m^2^). Subjects with BMI >25 kg/m^2^ were identified as overweight, while a BMI >30 kg/m^2^ was categorized as obese. Blood samples were collected in order to measure the fasting blood sugar (FBS) early in the morning after overnight fast for more than 10 h and post-prandial blood glucose (PPBG) levels after 2 h of the FBS.

### DNA extraction and genotyping

Genomic DNA was extracted from blood samples stored in EDTA using previously described methods (20). The DNA was dissolved in Tris–EDTA buffer at 100 ng/μL and stored at −80°C prior to the molecular analysis. C677T (*MTHFR*) and G1691A (*FVL*) mutations were screened using PCR and restriction fragment length polymorphism analysis performed with previously published primers and restriction enzymes (21) (Table 1). PCR amplification conditions were as follows; 35 cycles of denaturation at 95°C for 5 min, followed by annealing at 68°C for 30 s (C677T) or 56°C for 30 s (G1691A), and extension at 72°C for 5 min. PCR products were digested with appropriate restriction enzymes in a total volume of 20 μL for 16 h at 37°C, and analyzed by 2.5% agarose gel electrophoresis with ethidium bromide staining.

**Table 1:** **Details for genotyping *MTHFR* and *FVL* genes**.

Gene	SNP location	rs no.	Amino acid substitution	Forward primer	Reverse primer	Fragment	Annealing temperature	Enzyme
MTHFR	Exon 5	rs1801133	Ala 222 Val	TGAAGGAGAAGGTGT CTGCGGGA	GGACGGTGCGGTG AGAGTG	198 bp	68°C	*Hin*fI
FVL	Exon 10	rs6020	Arg 506 Gln	TCAGGCAGGAACAA CACCAT	GGTTACTTCAAGGACAAAA TACCTGTAAAGCT	241 bp	56°C	*Hin*dIII

### Statistical analysis

Hardy–Weinberg equilibrium was calculated in the cases and controls as described in our prior study (22). Differences between genotype/allele frequencies of GDM and non-GDM subjects were calculated using *T* test. The Yates correction was applied prior to analyzing genotype frequencies of *MTHFR* and *FVL* mutations. The odds ratio (OR) for genotype/phenotype relations and its 95% confidence interval (CI) was calculated performing chi-square test in the cross-tabs procedure for a 2 × 2 tables using SPSS software. All statistical analyses were performed using SPSS version 19.0 (SPSS Inc., Chicago, IL, USA).

## Results

### Characteristics of the study population

The clinical characteristics of GDM patients and non-GDM subjects are listed in Table 2. GDM cases (*n* = 137) were aged 22–38 years with a mean age of 26.7 ± 5.1 years, whereas the age and mean age for controls (*n* = 137) were 17–34 and 24.6 ± 3.55 years. The pre-pregnancy BMI range was 19.8–35.6 kg/m^2^ (mean: 26.8 ± 3.93 kg/m^2^) for GDM and 19–31.1 kg/m^2^ (mean: 24.1 ± 3.55 kg/m^2^) for controls (*p* = 0.28); 40.9% of individuals managed the diabetes with appropriate diet therapy and exercise, while 59.1% of patients required four to eight units of insulin therapy over the entire prenatal period. Age, weight, FBS, and PPBG values differed significantly between GDM cases and controls (*p* < 0.05).

**Table 2:** **Clinical details of GDM cases and controls**.

S. No.	Factors	GDM (*n* = 137)	Controls (*n* = 100)	*p* Value^a^
1	Age (years)	26.7±5.1	24.6±3.55	0.0001
2	BMI (kg/m^2^)	26.8±3.93	24.1±3.55	0.28
3	Weight (kg)	69.3±10.18	51.2±6.26	0.0001
4	Mean gestational age (weeks)	24.4±5.0	NA	NA
5	FBS (mg/dL)	110.6±3.93	99.24±11.37	<0.0001
6	PPBG (mg/dL)	158.80±47.76	112.00±39.70	0.05
7	Family history	80 (58.4%)	56 (56 %)	0.66
8	Insulin/diet therapy (*R*_x_)	81 (59.1%)/56 (40.9%)	NA	NA

*^a^t-Test applies to calculate the *p* value between GDM and non-GDM subjects*.

*NA, not analyzed/not applicable, BMI, body mass index; FBS, fasting blood sugar; PPBG, post-prandial blood glucose*.

### C677T allele and genotype frequencies

**Figure 1:**
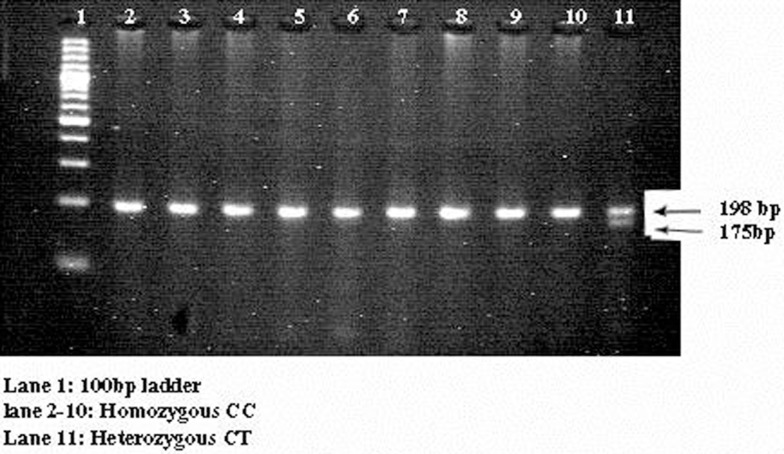
**Three percent agarose gel consist of digested products of C677T mutation**. It represents the 3% agarose gel picture. Lane 1 consists of 100 bp ladder. Lanes 2–10 consist of homozygous CC genotypes and lane 11 consists of heterozygous CT genotype.

Genotype and allele frequencies were in Hardy–Weinberg equilibrium in both groups. The PCR product encompassing the C677T mutation was 198 bp in length; digestion with *Hin*fI (G^↓^ANTC) yielded 198 and 175/23 bp fragments representing C and T alleles, respectively (Figure 1). Genotype frequencies for C677T CC, CT, and TT among GDM cases were 81.8, 18.2, and 0%, respectively. The frequencies of the T and C alleles were 9 and 91%. In control subjects, the frequencies of the CC, CT, and TT genotypes were 82, 18, and 0%, respectively, and C and T allele frequencies were 91 and 9%. Table 3 presents just the distribution of genotypes and alleles by GDM status. There was no evidence of a disease association for any of the allelic or genotype classes (OR: -1.015; 95% CI = 0.5378–1.916; *p* = 0.8892 and OR: -1.011; 95% CI = 0.5211–1.961; *p* = 0.9746).

**Table 3:** **Allele and genotype frequencies in GDM cases and controls for *MTHFR* gene**.

Genotype and allele	GDM cases (*n * = 137)	Controls (*n * = 100)	*p* Value^a^
*MTHFR* (rs1801133)

	***N*** (%)	***N*** (%)	

CC	112 (81.8)	82 (82)	0.96^a^
CT	25 (18.2)	18 (18)	
TT	0 (0)	0 (0)	
C	249 (0.09)	182 (91)	0.96^a^
T	25 (0.09)	18 (9)	

*^a^Chi-square *p* value*.

### G1691A allele and genotype frequencies

**Figure 2:**
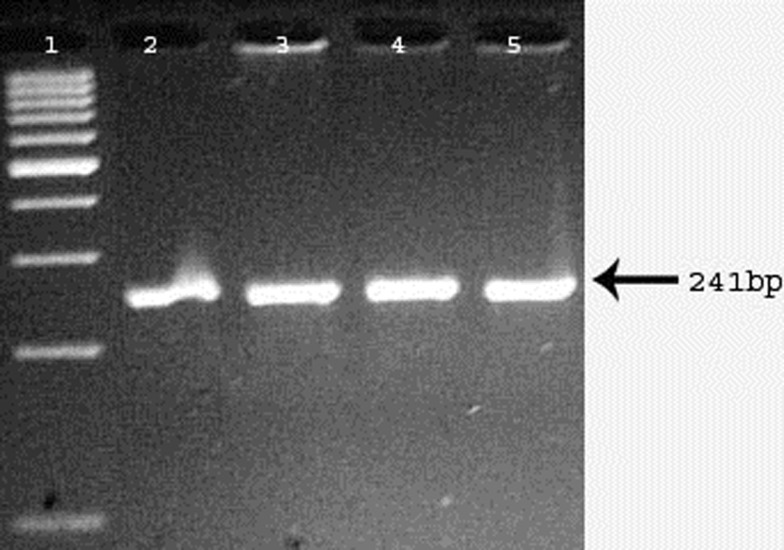
**Digested products appeared on 3% agarose gel for evaluation of FVL (G1691A) mutation**. Digested PCR products run on 3% agarose gel. Lane 1 represents 100 bp DNA ladder. Lanes 2–5 represent homozygous GG genotypes.

A 241-bp PCR product encompassing the G1691A mutation was digested with *Hin*dIII (A^↓^AGCTT), yielding a 209-bp fragment indicating the presence of the A allele (Figure 2). Only the GG (100%) genotype was observed in both cases and controls; therefore, this locus was not further analyzed.

## Discussion

This study investigated whether C677T and G1691A mutations are associated with the development of GDM among individuals of South Indian descent by genotyping age-matched cases and pregnant non-GDM subjects. The results did not reveal any association between investigated mutations and GDM risk in this study population.

Hyperglycemia is associated with an increased risk of unfavorable outcome in adults (T2DM), children (T1DM), and pregnant women (GDM) (23). *MTHFR* and *FVL* gene mutations are considered risk factors for hereditary thrombophilia and may influence the development of complications during pregnancy (24). We sought to determine whether C677T and G1691A mutations are associated with altered glucose levels during pregnancy, which could indicate GDM risk. Cases and controls differed significantly in terms of age, weight, FBS, and PPBG, but not with respect to genotype frequencies at either locus. These results suggest that the C677T and G1691A mutations are not involved in the development of GDM during pregnancy in Indian population.

The frequency of *MTHFR* gene mutations varies across geographical and ethnic groups. For example, the frequency of C677T in T2DM cases fluctuates between 40 and 49%, depending on the ethnicity of the study cohort (India, 40%; Turkey, 49%; Tunisia, 45%; Brazil, 46%; and China, 44%) (25–29). The C677T genotype frequencies (CC and CT) between north (25) and South Indian populations in T2DM and GDM subjects are varied. Both environmental and genetic factors contribute to the development and progression of GDM during pregnancy; this work showed that *MTHFR* mutations have no role in GDM in South Indian population.

The prior report from Australian population confirmed that 16.6% women harboring the *MTHFR* C677T gene mutation developed at least one pregnancy complication; however, the effect was restricted to women with small for gestational age (SGA) infants. There were no differences in genotype distribution among women with intrauterine fetal death, pre-eclampsia, or preterm delivery, and the study concluded that the mutation was a genetic marker for identifying women who are at increased risk of having an SGA infant (30).

Diabetes is a risk factor for thrombotic events (31) and atherothrombosis (32). The pathogenesis of diabetes involves hypercoagulability of the blood due to several factors such as the non-enzymatic glycosylation of clotting inhibitors. Patients who are carriers of a thrombophilic gene variant (e.g., factor V A1691G and∖or prothrombin A20210G) or other thrombotic risk factors are susceptible to the inherited or acquired thrombophilia. *FVL* mutations lead to a thrombophilic condition that is heritable both in hetero- and homozygous forms (33). Several previous studies have investigated a potential relationship between *FVL* gene mutations and diabetes incidence, and there is one report of a possible co-segregation of *FVL* and T2DM risk alleles (34).

A prevalence of 4.6% for the A allele of *FVL* G1691A (rs6020) has been reported among coronary artery disease patients with T2DM (35). In a later study, these authors reported prevalence rates for this mutation of 3.2, 1.6, and 4.9% among Iranian T2DM patients, T2DM patients with microalbuminuria, and normoalbuminuric T2DM patients, respectively, suggesting that the *FVL* G1691A mutation is not associated with microalbuminuria in this population (14). This mutation has also been implicated in the pathogenesis of thrombosis (36).

No association was found between FVL mutations and T2DM among Lebanese (36), Dutch (37), Japanese (38), and Caucasian patients (39).

In conclusion, *MTHFR* (C677T) and *FVL* (G1691A) mutations are not likely to be genetic risk factors for the development of GDM in South Indian women.

## Conflict of Interest Statement

The authors declare that the research was conducted in the absence of any commercial or financial relationships that could be construed as a potential conflict of interest. The Review Editor Suresh Kumar Chitta declares that, despite having collaborated on a publication in the last 2 years with author Noor Ahmad Shaik, the review process was handled objectively.
